# Effect of Wheat Flour Substitution with Medicinal Mushroom Powder on Protein and Starch Digestibility and Functional Properties of Bread

**DOI:** 10.3390/molecules30224380

**Published:** 2025-11-13

**Authors:** Paulina Łysakowska, Aldona Sobota, Małgorzata Gumienna

**Affiliations:** 1 Department of Engineering and Cereal Technology, University of Life Sciences in Lublin, Skromna 8, 20-704 Lublin, Poland; paulina.lysakowska@up.edu.pl; 2 Department of Food Technology of Plant Origin, Poznań University of Life Sciences, Wojska Polskiego 31, 60-624 Poznan, Poland; malgorzata.gumienna@up.poznan.pl

**Keywords:** medicinal mushrooms, functional bread, protein digestibility, resistant starch, β-glucans, in vitro digestion

## Abstract

Medicinal mushrooms are recognised as a source of bioactive polysaccharides and proteins with potential health benefits. This study presents the first comparative evaluation of wheat bread fortified with powdered fruiting bodies of three medicinal mushroom species: *Hericium erinaceus* (Lion’s Mane), *Ganoderma lucidum* (Reishi), and *Ophiocordyceps sinensis* (Cordyceps). Wheat flour was partially substituted at levels of 3–12%, and the resulting breads were analysed for (1→3)(1→6)-β-D-glucan content as well as in vitro protein and starch digestibility. Mushroom enrichment significantly increased β-glucan concentration in bread, with the greatest enhancement observed for 12% Reishi substitution (5.67% d.m.). Starch digestibility decreased across all fortified breads, accompanied by a substantial increase in resistant starch, particularly for Reishi bread (+427% relative to control). Protein digestibility was also reduced, most prominently in Cordyceps bread (−32.7 percentage points), although these products still provided up to ~52% more total protein than the control. The results confirm that incorporating medicinal mushrooms into wheat bread effectively increases its content of bioactive components, such as β-glucans, resistant starch and protein, indicating its potential as a nutrient-enriched bakery product with improved functional value.

## 1. Introduction

Modern food systems are rapidly evolving in response to consumer demand for products with scientifically validated health benefits. This trend stems from the growing prevalence of metabolic disorders, including obesity and type 2 diabetes, and the need to enhance overall dietary quality [1,2,3]. Within this framework, functional foods enriched with bioactive compounds capable of modulating carbohydrate metabolism, immune responses, and gut microbiota composition have gained particular attention [4,5].

Medicinal mushrooms, particularly *Hericium erinaceus* (Bull.) Pers. (Lion’s Mane), *Ganoderma lucidum* (Curtis) P. Karst. (Reishi), and *Ophiocordyceps sinensis* (Berk.) G.H. Sung, J.M. Sung, Hywel-Jones & Spatafora (Cordyceps), represent valuable sources of bioactive polysaccharides, triterpenoids, and proteins exhibiting metabolic activity [6,7,8]. Among these compounds, fungal β-glucans are of particular importance. They are polysaccharides composed of β-(1→3)-linked glucose backbones with β-(1→6) branches, and their bioactivity depends on molecular weight, branching degree, and solubility, which vary among fungal species and cultivation conditions [9,10,11,12]. Reported β-glucan contents range from 4.3 to 23.6% d.m. in *G. lucidum*, 3.8–35.3% d.m. in *H. erinaceus*, and around 3.8% d.m. in *O. sinensis* [8], exceeding the values typically observed in cereal β-glucans such as those from oats or barley [13,14].

Bread, as a widely consumed staple food, offers an effective matrix for the incorporation of bioactive ingredients [15]. However, the introduction of mushroom powders into bread formulations adds insoluble fungal cell-wall polysaccharides that can modify protein and starch digestibility. In conventional wheat bread, starch digestibility reaches approximately 86%, and protein digestibility about 96% [16]. Increased fibre content can restrict enzymatic hydrolysis through steric hindrance and altered hydration [17], which may reduce postprandial glycaemic and insulinaemic responses and thereby support metabolic regulation [18,19]. Undigested carbohydrate fractions, including resistant starch (RS), undergo microbial fermentation in the colon to produce short-chain fatty acids (SCFAs) such as acetate, propionate, and butyrate, which contribute to improved gut and cardiometabolic health [20,21]. Consequently, reduced nutrient digestibility in such formulations may represent a beneficial nutritional modification rather than a technological limitation [22].

While several studies have explored the effects of medicinal mushrooms in bakery products, most have investigated a single species or focused primarily on physicochemical and sensory attributes rather than nutrient bioaccessibility. To date, no comparative study has simultaneously evaluated three medicinal mushroom species with respect to their influence on starch and protein digestibility, while quantitatively linking these effects to β-glucan content in a cereal matrix.

This research provides a novel comparative perspective on the structure–function relationships between fungal β-glucans and macronutrient digestibility in bread. The working hypothesis posits that mushroom-derived β-glucans, due to their water-binding capacity and viscosity-enhancing properties, reduce enzymatic accessibility of starch and proteins within the wheat-based product. Substitution levels of 3–12% were chosen based on previous sensory and technological assessments to maintain acceptable bread quality while enabling evaluation of dose-dependent effects.

The objective of this study was to determine the impact of *H. erinaceus*, *G. lucidum*, and *O. sinensis* powders on β-glucan content, water absorption properties (WAI), water solubility index (WSI), starch and protein digestibility, and resistant starch content in wheat bread. The results provide new insight into the development of functional bakery products and their potential role in dietary strategies aimed at supporting metabolic health.

## 2. Results and Discussion

### 2.1. Water Solubility Index (WSI) and Water Absorption Index (WAI)

Substitution of wheat flour with mushroom powders at levels of 3–12% resulted in a significant (*p* ≤ 0.05) increase in the WAI of bread (Table 1). The highest increase (11.6% compared to the control) was observed with 12% substitution using Reishi powder, which may be attributed to the higher content of components with strong water-binding capacity, such as β-glucans and chitin in this mushroom [23,24]. Smaller but still noticeable increases were recorded at the 12% substitution level with Lion’s Mane (+7.7%) and Cordyceps (+7.4%). A similar effect was reported by Mandliya et al. [25], who demonstrated that dried mycelium of *Pleurotus eryngii* enhanced the WAI of bread due to the presence of fibre fractions with high water-binding capacity.

The WSI of bread depended both on the mushroom species and the substitution level. The greatest increase in WSI (19.4% relative to the control) was recorded for breads with 12% substitution with Cordyceps powder. It should be noted that Cordyceps itself exhibited considerably higher solubility of dry matter compared with Lion’s Mane and Reishi, which can be explained by the presence of water-soluble low-molecular-weight compounds, such as sugars and amino acids. Due to their low molecular weight and hydrophilicity, these compounds readily diffuse into the aqueous phase, and their presence in water extracts of Cordyceps has was confirmed by He et al. [26].

This effect corresponds to the observations of Zhang et al. [27], who reported that the addition of *Cordyceps militaris* powder enhanced the extractability of components from wheat dough. A moderate increase in WSI was observed for Lion’s Mane (+8.7%), while the change recorded for Reishi was marginal (+0.4%), which may indicate the predominance of water-insoluble compounds in the fruiting bodies of this species. A similar phenomenon was described by Hitayezu and Xiong [28], who showed that finer fractions of *Shimeji* powder, despite their greater water retention capacity, did not exhibit higher solubility of dry matter. Comparable conclusions were also drawn by Uukule et al. [29], who demonstrated that the addition of oyster mushroom flour increased WSI without affecting WAI, which was attributed to the presence of soluble mycelial fractions.

### 2.2. Protein Digestibility

**Figure 1:**
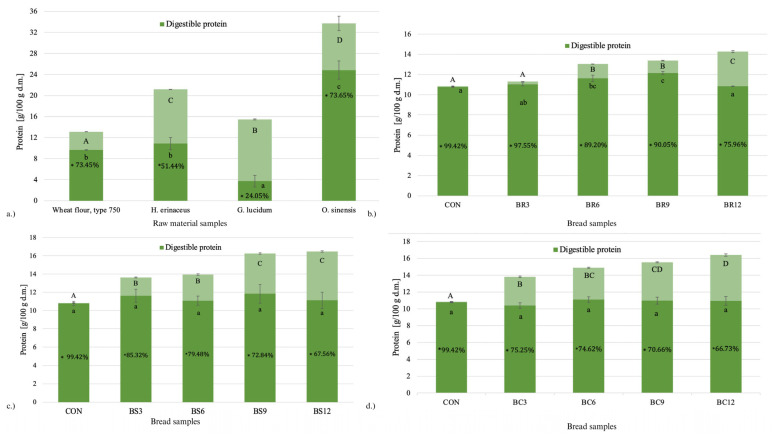
Total and digestible protein content (% d.m.) in raw materials (**a**) and wheat breads enriched with *Ganoderma lucidum* (Reishi; BR3–BR12) (**b**), *Hericium erinaceus* (Lion’s Mane; BS3–BS12) (**c**), and *Ophiocordyceps sinensis* (Cordyceps; BC3–BC12) (**d**) at different wheat flour substitution levels (3–12%). CON—control bread without mushroom addition. Bars represent mean values ± standard deviation (*n* = 3). Light green segments indicate total protein content, and dark green segments represent digestible protein fractions. Protein digestibility values (%) are marked with an asterisk (*). Different uppercase letters denote statistically significant differences in total protein content between samples within each subplot (*p* ≤ 0.05; Tukey’s test), while lowercase letters indicate significant differences in digestible protein content.

The protein content and digestibility of the tested raw materials differed significantly (*p* ≤ 0.05) (Figure 1). All mushroom powders were characterised by a higher total protein content compared to wheat flour, with the highest levels observed in *O. sinensis* powder and the lowest in *G. lucidum*. Similarly, the greatest amount of digestible protein was recorded in *O. sinensis* (approximately 2.6-fold higher compared with wheat flour), whereas *G. lucidum* showed the lowest availability of protein to proteolytic enzymes. *H. erinaceus* powder contained about 61% more protein than wheat flour and showed only a slightly higher digestibility (+13%). It should be emphasised, however, that the low protein digestibility determined in raw materials may not directly reflect protein digestibility in bread. Yeast fermentation enhances proteolysis, leading to increased protease activity and improved protein digestibility, while thermal processing during baking may inactivate natural protease inhibitors and induce protein structural modifications, thereby increasing their susceptibility to enzymatic hydrolysis [30,31,32].

Substitution of wheat flour with medicinal mushroom powders in bread formulation had a significant effect (*p* ≤ 0.05) on reducing protein digestibility (Figure 1b–d). The control sample (CON), without mushroom addition, showed the highest digestibility (99.42%), exceeding the value determined for pure type 750 wheat flour (73.45%). This observation can be attributed to favourable changes in protein structure during baking, including thermal denaturation, inactivation of natural protease inhibitors, and modifications in the gluten matrix that improve substrate accessibility to proteolytic enzymes.

The addition of mushroom powders resulted in a marked reduction in protein digestibility, with the extent depending on both the mushroom species and the substitution level. The smallest changes were noted in bread with *G. lucidum* (Figure 1b), where digestibility decreased by 1.86 percentage points at 3% substitution and by 23.5 percentage points at 12%, relative to the control. This phenomenon may be explained by the high concentration of structural components in Reishi, such as chitin and β-glucans, which can hinder the accessibility of proteolytic enzymes [33,34].

In breads enriched with *H. erinaceus* (Figure 1c), a reduction in protein digestibility compared to the control was also observed, with a decrease of up to 31.9 percentage points at 12% substitution. However, the strongest negative impact was exerted by *O. sinensis*. Even at 3% substitution, digestibility declined by more than 24 percentage points, while at 12% substitution the reduction reached 33 percentage points. This substantially lowered the biological value of protein in breads containing *O. sinensis*, despite its favourable amino acid profile [35]. The markedly stronger reduction in protein digestibility observed in breads enriched with *O. sinensis* may also relate to the presence of cordycepin (3′-deoxyadenosine), which has been shown to interfere with ATP-dependent proteolytic pathways and inhibit protein synthesis in biological systems through modulation of mTOR and AMPK signalling [36,37,38]. Although such effects have not yet been verified in cereal-based matrices, a potential modulation of protease activity within mushroom-enriched dough cannot be excluded and warrants further investigation.

It has been confirmed that *G. lucidum*, *H. erinaceus* and *O. sinensis* contain chitin, which, together with other polysaccharides, contributes to their biological properties [39]. The presence of this biopolymer may also limit protein digestibility. A similar phenomenon was described for insect proteins, where chitin removal from black soldier fly larvae (*Hermetia illucens*) significantly improved solubility and digestibility [40]. Chitin derivatives such as chitosan have also been shown to negatively affect the digestibility of whey protein isolates by precipitating proteins and modulating protease activity [41]. Conversely, partial hydrolysis of chitin using lysozymes can be beneficial, improving the quality of insect-protein-enriched bread by increasing loaf volume, softness, and sensory acceptability [42].

Partial substitution of wheat flour with *H. erinaceus* increased digestible protein content by approximately 3–10% relative to the control, with the highest effect observed at 9% substitution. A similar trend was found for *G. lucidum*, where digestible protein content increased by about 2–12%, also peaking at 9% substitution. In the case of *O. sinensis*, the increase in digestible protein was notably weaker.

The interpretation of reduced protein digestibility must consider both nutritional and functional aspects. Medicinal mushrooms provide additional proteins and dietary fibre that may alter digestive behaviour of the cereal matrix without impairing sensory quality. Our previous work on breads supplemented with *H. erinaceus*, *G. lucidum*, and *O. sinensis* confirmed that substitution levels up to 9% maintained overall sensory acceptability and desirable crumb properties [43,44,45]. Therefore, the in vitro reduction in digestibility should not be considered detrimental but rather a potentially beneficial modification requiring further verification through in vivo studies assessing amino acid utilisation.

In this context, the findings of Tańska et al. [46] are of particular interest, demonstrating that powders from *Alphitobius diaperinus* (lesser mealworm) and *Acheta domesticus* (house cricket) enhanced protein content and improved the amino acid profile of shortbread cookies. Analogously, despite potentially limited protein bioavailability, mushrooms can serve as valuable sources of limiting amino acids in cereal-based products. Future research should therefore focus on strategies to improve the digestibility and bioavailability of mushroom-derived proteins, including optimisation of baking parameters, the use of protein isolates, or the application of additives capable of partial hydrolysis of compounds limiting protein accessibility.

### 2.3. Starch Digestibility

The incorporation of medicinal mushroom powders into wheat bread significantly influenced starch digestibility. With increasing substitution levels of *Hericium erinaceus*, *Ganoderma lucidum* and *Ophiocordyceps sinensis* (3–12%), a systematic reduction in the digestible starch (DS) fraction was observed. The most pronounced decrease occurred in breads fortified with Reishi; in BR12, DS content was approximately 23% lower compared with the control (CON). Moderately smaller reductions were recorded in BC12 (≈15%) and BS12 (≈12%). This effect is likely associated with insoluble fungal cell-wall polysaccharides, such as β-glucans and chitin, which form physical barriers around starch granules and limit both gelatinisation and enzymatic access, as well as with hydrogen-bonding interactions that reduce starch bioavailability [47,48]. Comparable findings were reported by Uukule et al. [29] in products fortified with *Pleurotus ostreatus*.

Although Reishi powder itself exhibited the highest intrinsic digestible starch (DS) content among the tested mushroom materials, bread fortified with *G. lucidum* (BR12) showed the lowest overall starch digestibility. This apparent discrepancy can be explained by physicochemical interactions occurring during dough fermentation and baking. The relatively high levels of β-glucans and chitin in *G. lucidum* may enhance matrix viscosity and promote the formation of coating and cross-linked structures that encapsulate starch granules, thereby limiting enzymatic accessibility after thermal processing [49]. Consequently, *G. lucidum* exerted a dual effect, simultaneously producing the largest reduction in digestible starch and the most pronounced increase in resistant starch (RS), indicating that its structural polysaccharides substantially influenced starch transformation and retrogradation within the bread matrix.

Feng et al. [50] showed that β-glucans from *H. erinaceus* enhance dough viscosity, reduce amylose leaching and physically coat starch granules, thereby decreasing enzyme accessibility to gelatinised starch. Similar steric hindrance and hydration-limiting effects may occur in Reishi-containing breads. Species-dependent variability in β-glucan structure likely explains why changes in DS and RS differed among mushrooms; however, detailed polysaccharide structural characterisation was outside the scope of this work. Further microscopic evaluation is required to confirm these structural influences within the wheat matrix.

The extent of digestibility reduction may also depend on the molecular characteristics of β-glucans. High-molecular-weight, low-solubility β-glucans form dense and viscous networks that restrict starch granule swelling and reduce α-amylase diffusion, thereby limiting hydrolysis efficiency [51,52,53]. In contrast, β-glucans of lower molecular weight and greater solubility provide weaker steric barriers and allow more efficient enzymatic penetration [54,55,56]. Although β-glucan physicochemical properties were not assessed here, differences in digestibility reductions among breads support the relevance of species-specific fungal cell-wall composition. Future studies incorporating polysaccharide molecular-weight profiling and SEM/CLSM imaging are required to confirm these proposed structure–function relationships.

The formation of RS was strongly dose-dependent. BR12 demonstrated the highest RS increase, exceeding 400% compared with the control. Significant, though less pronounced, increases were also noted in breads containing Lion’s Mane and Cordyceps. These changes may stem from both reduced enzymatic accessibility and altered physicochemical behaviour of starch, including enhanced retrogradation and formation of starch–lipid complexes [57,58]. RS escapes digestion in the small intestine and undergoes colonic fermentation into short-chain fatty acids (SCFAs), including butyrate, propionate and acetate, which exert beneficial physiological effects: butyrate supports colonocyte energy metabolism and improves intestinal barrier integrity; propionate influences glucose regulation; and acetate may modulate appetite and lipid metabolism [59,60]. Thus, the elevated RS levels may represent a favourable nutritional modification in these functional breads.

Nevertheless, interpretation of the current findings requires caution. Starch gelatinisation was not directly quantified and bread microstructure was not evaluated using SEM or CLSM techniques, which could have revealed potential changes in starch–protein network organisation. Additionally, no glycaemic index measurements or in vitro glucose-release kinetics were applied, preventing conclusions regarding postprandial glycaemic responses. Therefore, mechanistic explanations remain hypothetical and should be validated in future studies integrating molecular characterisation with in vivo digestibility assessment.

Total starch digestibility (SD), expressed as the ratio of DS to total starch content, also decreased significantly, although to a lesser extent compared with DS alone. The greatest reduction was observed in BR12 (nearly six percentage points below CON), while breads containing the remaining mushrooms exhibited milder changes. Even slight structural alterations within starch granules, variations in amylose/amylopectin chain organisation or differences in gelatinisation behaviour can substantially influence amylolysis rates and final starch digestibility [61,62] (Table 2).

### 2.4. β-Glucan Content

**Figure 2:**
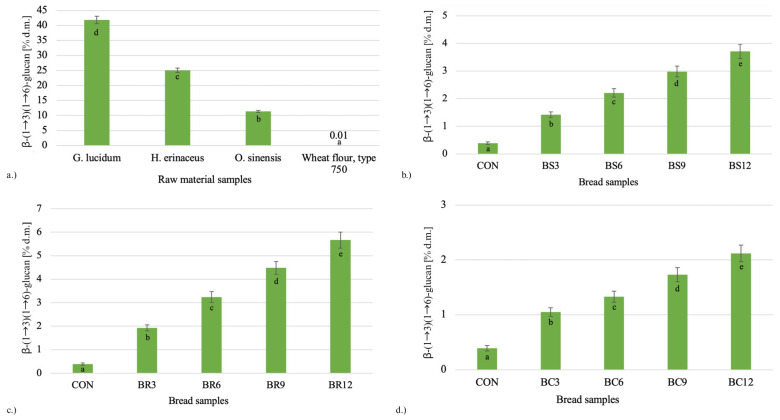
Content of β-(1→3)(1→6)-D-glucans in (**a**) raw material samples and in wheat breads enriched with (**b**) *Hericium erinaceus* (Lion’s Mane), (**c**) *Ganoderma lucidum* (Reishi), and (**d**) *Ophiocordyceps sinensis* (Cordyceps) powders at different levels of wheat flour substitution (3–12%). Data are expressed as mean ± standard deviation (*n* = 3). Different lowercase letters (a–e) displayed on the bars indicate statistically significant differences among samples within each graph (*p* ≤ 0.05; Tukey’s test). Sample codes: CON—control bread without mushroom addition; BS3–BS12—breads with *H. erinaceus* powder (3–12% substitution); BR3–BR12—breads with *G. lucidum* powder; BC3–BC12—breads with *O. sinensis* powder.

β-glucans are polysaccharides present in fungal cell walls, playing a key role in their structure and metabolic functions. Due to their beneficial health-promoting properties, including immunomodulatory activity and cholesterol-lowering effects, β-glucans are considered valuable functional components. The higher β-glucan content observed in wheat bread compared with wheat flour (Figure 2) may be linked to the use of Saccharomyces cerevisiae during dough fermentation, as β-glucans are also present in the cell wall structure of these yeasts. According to literature data, the dry mass of S. cerevisiae cells contains up to 12% β-glucans, while in purified form their concentration may reach as high as 76.56% [63].

The efficiency of bread enrichment with β-glucans was determined by the natural content of these compounds in the mushroom powders. The highest β-glucan levels were found in Reishi (41.8% d.m.), moderate levels in Lion’s Mane (25.1% d.m.), and the lowest in Cordyceps (11.4% d.m.) (Figure 2). Consequently, the greatest increase in β-glucan content was recorded in bread enriched with Reishi. Substitution of 12% wheat flour with this powder resulted in more than a fourteenfold increase in β-glucan content in the bread sample. Analogous substitution with Lion’s Mane (BS12) and Cordyceps (BC12) led to approximately nine- and fivefold increases, respectively.

These findings confirm that the variability in bioactive polysaccharide content of the applied mushroom components directly translates into their levels in final products. The very high β-glucan content observed in Reishi is consistent with literature reports highlighting the considerable potential of this species in the development of novel functional foods [8,64].

The increase in β-glucan content in mushroom-enriched breads may also explain the previously observed effects of reduced protein and starch digestibility and increased resistant starch content, particularly in Reishi bread. According to the literature, fungal β-glucans can form physical barriers around starch granules, limiting the access of amylolytic enzymes and increasing the viscosity of the food matrix, thereby affecting the rate of starch hydrolysis [21,64]. In addition, β-glucans from different mushroom species differ in branching degree, conformation and solubility, which may influence the extent of steric hindrance imposed on starch during enzymatic hydrolysis, providing a plausible explanation for the stronger reduction in starch digestibility observed for Reishi bread [65,66]. Although these structural characteristics were not analysed in this study, the lack of SEM/CLSM microstructural data represents a limitation and should be addressed in future work to directly visualise starch granule coating or gluten matrix modification in mushroom-enriched breads.

**Figure 3:**
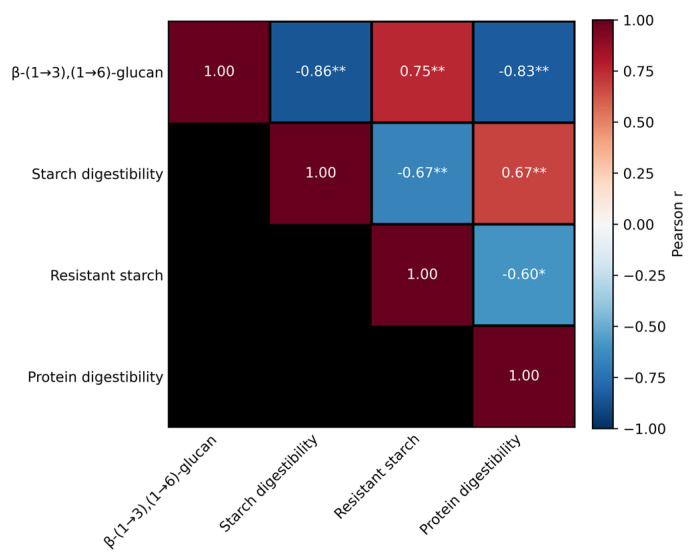
Pearson’s correlation matrix illustrating relationships between β-(1→3)(1→6)-D-glucan content, starch digestibility, resistant starch, and protein digestibility in wheat breads enriched with medicinal mushroom powders. Positive correlations are shown in red and negative correlations in blue. The intensity of colour corresponds to the magnitude of the correlation coefficient (*r*). Asterisks indicate levels of statistical significance: *p* ≤ 0.05 (*), *p* ≤ 0.01 (**).

This mechanism was statistically supported by Pearson’s correlation analysis (Figure 3), which revealed strong negative correlations between β-glucan content and both starch and protein digestibility (*p* ≤ 0.01), along with a significant positive correlation with resistant starch (*p* ≤ 0.01). Moreover, the positive relationship between starch and protein digestibility, combined with their negative association with resistant starch (*p* ≤ 0.05), further substantiates the integrated influence of β-glucans on nutrient digestibility and enzymatic accessibility within the bread matrix.

An increasing number of studies also indicate the significant immunological properties of fungal β-glucans. For example, Case et al. [65] demonstrated that polysaccharide fractions isolated from *Agaricus bisporus* could activate a “trained immunity” response via the Dectin-1 receptor, enhancing innate immunity and improving resistance to oxidative stress and infection. Moreover, fungal β-glucans may modulate gut microbiota composition by promoting the growth of beneficial bacteria such as Bifidobacterium and Lactobacillus. In in vitro fermentation studies, β-glucan isolated from shiitake was shown to support the growth of fibre-fermenting bacteria while increasing butyrate and propionate production [66].

Increased β-glucan content also has technological implications. Due to their water-binding and viscosity-enhancing properties, β-glucans can influence dough rheology, gas retention, and crumb structure during baking. Our previous studies demonstrated that breads enriched with *G. lucidum* remained sensorially acceptable up to 6% substitution, while higher levels (≥9%) were associated with a decline in overall flavour scores (*p* ≤ 0.05). Formulations with *O. sinensis* were acceptable up to 12%, whereas *H. erinaceus* led to a perceptible decline in acceptability only at the upper concentration range (9–12%) [35,36,37,38,39,40,41,42,43]. These findings indicate that β-glucan enrichment should be optimised to balance functional enhancement with sensory quality in future product development.

## 3. Methodology

### 3.1. Plant Material

The study utilised wheat flour type 750 (Polskie Młyny, Warsaw, Poland), characterised by the following parameters: ash content—0.74% d.m., wet gluten content—27.5% ± 1.0, gluten index—99.0 ± 0.3, falling number—304 s ± 6, and mean particle size—0.12 mm.

As a functional additive, powdered fruiting bodies of three medicinal mushroom species were applied: Lion’s Mane (*Hericium erinaceus*), Reishi (*Ganoderma lucidum*), and Cordyceps (*Ophiocordyceps sinensis*) (NatVita, Mirków, Poland). All raw materials were stored in sealed, opaque containers at temperatures below 25 °C and relative humidity of 60–65% until further use.

### 3.2. Chemicals

A Yeast and Mushroom β-Glucan Assay Kit (K-YBGL) and a Resistant Starch Assay Kit (K-RSTAR) were purchased from Megazyme (Neogen Corporation, Lansing, MI, USA). The following enzymes were also used: pepsin (from porcine gastric mucosa), pancreatin (from porcine pancreas), amyloglucosidase, invertase, trehalase, exo-1,3-β-glucanase, and β-glucosidase, all obtained from Sigma-Aldrich (St. Louis, MO, USA).

Chemical reagents employed in the analyses included: sulphuric acid (H_2_SO_4_), sodium hydroxide (NaOH), trichloroacetic acid (TCA), sodium azide, phosphate buffers, and the GOPOD reagent (containing glucose oxidase, peroxidase, and 4-aminoantipyrine), supplied by Sigma-Aldrich. HPLC-grade reagents, including acetonitrile and formic acid, were purchased from Merck (Darmstadt, Germany). Analytical standards of glucose and protein were used for calibration of quantitative assays. All other chemicals applied in the study were of analytical grade.

### 3.3. Bread Production

Bread formulation consisted of wheat flour or flour–mushroom powder mixture 100% (600 g), fresh yeast 3% (18 g), salt 1.5% (9 g), and the amount of water determined by farinograph absorption (AACC 54-21) as reported previously [43,44,45]. The dough was mixed using a BEAR Varimixer Teddy 5 L (Varimixer A/S, Copenhagen, Denmark): 3 min at low speed followed by high speed until gluten development. Fermentation was conducted for 90 min at 30 °C and 85 ± 2% RH (Tefi Klima Pro 100, Debag, Bautzen, Germany), with degassing after 60 min. Next, the dough was divided into 290 ± 5 g portions, manually shaped, placed into baking tins (18 × 7.5 × 7.0 cm), and proofed for 30 min under the same conditions. Baking was performed in a Helios Pro 100 oven (Debag, Bautzen, Germany) at 230 °C for 30 min. Three independent baking batches were produced for each formulation (biological replicates, *n* = 3). Three loaves from each batch were dried, milled, and combined into a composite sample for analysis. All stages of dough preparation, fermentation, and baking followed standardised laboratory conditions consistent with our previous works [43,44,45], ensuring process reproducibility.

### 3.4. Water Solubility Index (WSI) and Water Absorption Index (WAI)

The water solubility index (WSI) and water absorption index (WAI) were determined according to the procedure of Zarzycki et al. [67], with modifications adapted to the characteristics of the tested samples. A portion of dried and ground bread (2 g, d.m.) was mixed with 30 mL of distilled water (20 °C) in centrifuge tubes (50 mL), shaken, and incubated for 15 min at room temperature. The suspensions were then centrifuged (15 min, 2200× *g*). An aliquot of the supernatant (10 mL) was dried at 105 °C to constant weight. Based on this, WSI (% d.m.) was calculated as the ratio of the dry mass of the residue after evaporation (W_ds_) to the dry mass of the sample (W_dm_), according to the following equation:

(1) $$\mathrm{WSI}\;=\;\frac{W_{ds}}{W_{dm}}\cdot 100\%$$

WAI was calculated as the ratio of the gel mass after centrifugation (W*_g_*) to the dry mass of the sample (W*_dm_*):

(2) $$\mathrm{WAI}\;=\;\frac{W_{g}}{W_{dm}}\cdot 100\%$$

Results are expressed on a dry matter basis as mean ± SD. Three biological replicates were pooled prior to analysis, which was performed in triplicate.

### 3.5. Determination of Protein Digestibility

Protein digestibility was determined in vitro using pepsin and pancreatin, according to the procedure described by Saunders et al. [68]. Water and pepsin dissolved in 0.1 N HCl were added to the samples, which were then incubated at 37 °C for 3 h. After this stage, the samples were neutralised, supplemented with pancreatin dissolved in phosphate buffer (pH 8) and sodium azide (0.005 M), and further incubated at 37 °C for 24 h. Upon completion of protein hydrolysis, 45% trichloroacetic acid (TCA) solution was added to precipitate proteins, followed by centrifugation at 5500× *g*. Protein content in the supernatant was determined using the Kjeldahl method in accordance with ISO 20483 [69]. Protein digestibility was expressed as the percentage of digested protein relative to the total protein content in the sample (% protein digestibility). Total protein content in the analysed samples was determined using the Kjeldahl method, following ISO 20483. Protein digestibility was calculated on a dry matter basis. Results are expressed as mean ± SD. Three biological replicates were pooled prior to analysis, which was performed in triplicate.

### 3.6. Determination of Starch Digestibility

Starch digestibility was determined using an enzymatic method with the Resistant Starch Assay Kit (K-RSTAR, Megazyme, Neogen Corporation, Lansing, MI, USA), according to the recommended protocol (version 08/23, AOAC 2002.02; AACC 32-40.01). Samples (100 mg) were incubated with pancreatic α-amylase and amyloglucosidase at 37 °C for 16 h, resulting in the hydrolysis of the digestible starch fraction to glucose. The mixture was then centrifuged, and the supernatant containing solubilised starch was collected for further analysis. Resistant starch was separated by precipitation and repeated washing with 50% ethanol, then dissolved in 2 M KOH, neutralised, and subjected to complete hydrolysis using amyloglucosidase.

The amount of released glucose was determined colorimetrically using a spectrophotometer (Thermo Electron, Waltham, MA, USA) after reaction with GOPOD reagent (glucose oxidase/peroxidase and 4-aminoantipyrine), measuring absorbance at 510 nm. The total starch content was calculated as the sum of digestible starch (solubilised starch) and resistant starch, each converted from glucose to anhydroglucose using the factor 162/180. Starch digestibility was expressed as the ratio of digestible starch to total starch, according to the following equation:

(3) $$Starch\;digestibility\;(\%)=\frac{Digestible\;starch\;(\frac{g\;}{100g\;d.m.}\;sample)}{Total\;starch(\frac{g}{100g\;d.m.}\;sample)}$$

Starch digestibility and resistant starch content were calculated on a dry matter basis. Results are expressed as mean ± SD. Three biological replicates were pooled prior to analysis, which was performed in duplicate. Calibration curves for glucose quantification showed high linearity (R^2^ ≥ 0.999), and spike-and-recovery tests resulted in recoveries between 98% and 102%.

### 3.7. Determination of β-Glucan Content

The β-glucan content was determined using the Yeast and Mushroom β-Glucan Assay Kit (K-YBGL, Megazyme, Neogen Corporation, Lansing, MI, USA), following the manufacturer’s protocol (version 08/23). The method involved the quantification of total glucans (α- and β-glucans) and α-glucans, with β-glucan content calculated as the difference between total and α-glucan values.

Total glucans were measured after dissolution and partial hydrolysis of the samples (90 mg) in 12 M H_2_SO_4_, followed by further hydrolysis in 2 M H_2_SO_4_. Remaining oligosaccharides were enzymatically degraded using exo-1,3-β-glucanase and β-glucosidase. The released glucose was quantified colorimetrically using GOPOD reagent, with absorbance measured at 510 nm.

α-Glucans were determined in separate samples (100 mg), extracted with 1.7 M NaOH. After neutralisation and buffering, enzymatic hydrolysis was performed with amyloglucosidase, invertase, and trehalase. The liberated glucose was quantified colourimetrically with GOPOD reagent, as described for total glucans. β-(1→3)(1→6)-D-glucan content was calculated as the difference between total glucans and α-glucans, using a conversion factor from free glucose to anhydroglucose (162/180). Analyses were carried out in duplicate, and accuracy was verified against the results obtained for the control sample.

A glucose standard (100 µg) was analysed in each run to calculate a conversion factor for quantification. The average absorbance of the glucose standard was 1.0192 ± 0.0016, resulting in a factor of 98.12 µg glucose·Abs^−1^. Linearity of the assay was confirmed, with calibration curve R^2^ ≥ 0.999. β-Glucan content was expressed on a dry weight basis as mean ± SD. Three biological replicates were pooled prior to analysis which was performed in triplicate.

### 3.8. Statistical Analysis

All data were analysed using STATISTICA 13 (StatSoft, Tulsa, OK, USA). Results are presented as mean ± SD. Normality of data distribution was verified using the Shapiro–Wilk test, and homogeneity of variances using Levene’s test. One-way ANOVA was applied to determine significance, and Tukey’s post hoc test with *p*-value adjustment for multiple comparisons was used (*p* ≤ 0.05).

Pearson’s correlation coefficients (r) were calculated to assess the relationships between β-glucan content and digestibility parameters (starch digestibility, resistant starch, and protein digestibility). The statistical significance of correlation coefficients was evaluated at two levels: *p* ≤ 0.05 and *p* ≤ 0.01. In the graphical visualisation, statistically significant correlations were indicated by (*p* ≤ 0.05) and (*p* ≤ 0.01), while correlations with *p* ≥ 0.05 were treated as non-significant. The correlation matrix and heatmap were generated using the Python programming environment (Python 3.10.12) in Google Colaboratory (Google LLC, Mountain View, CA, USA) with the NumPy (1.26.4), Pandas (2.2.2), SciPy (1.13.1), and Matplotlib (3.9.2) libraries.

## 4. Summary

This study demonstrated that the incorporation of medicinal mushroom powders derived from *Ganoderma lucidum*, *Hericium erinaceus*, and *Ophiocordyceps sinensis* (3–12%) into wheat bread markedly altered its nutritional characteristics. The content of β-(1→3)(1→6)-D-glucans increased proportionally with the level of substitution, most prominently in the Reishi-enriched samples, and was strongly associated with reduced starch and protein digestibility, as well as higher content of resistant starch.

The findings indicate that mushroom powders can enhance the functional value of bread by increasing the proportion of health-relevant dietary fibre fractions while maintaining desirable macronutrient levels. The observed modifications highlight mushroom-enriched bread as a promising concept for functional food products aimed at supporting the prevention and management of diet-related metabolic disorders.

This study has certain limitations. Structural characterisation (e.g., SEM/CLSM microscopy) and glycaemic response testing were not performed; therefore, the proposed mechanisms remain hypothetical and require further experimental verification.

From an industrial perspective, the inclusion of mushroom powders is technologically feasible at moderate substitution levels and offers potential for clean-label innovation within the bakery sector. Further optimisation may facilitate scale-up towards commercial bread products enriched in fungal β-glucans.

## Figures and Tables

**Table 1:** Water absorption index (WAI) and water solubility index (WSI) of wheat bread enriched with medicinal mushroom powders.

Sample	WAI [% d.m.]	WSI [% d.m.]
Lion’s Mane	169.32 ^C^ ± 0.83	17.30 ^C^ ± 0.03
Reishi	213.08 ^D^ ± 1.23	13.23 ^B^ ± 0.14
Cordyceps	146.48 ^B^ ± 0.94	29.31 ^D^ ± 0.21
Wheat Flour type 750	119.26 ^A^ ± 0.39	08.25 ^A^ ± 0.07
CON	134.69 ^a^ ± 1.31	13.08 ^a^ ± 0.04
BS3	135.72 ^ab^ ± 0.24	13.23 ^ab^ ± 0.11
BS6	137.80 ^bc^ ± 1.42	13.35 ^b^ ± 0.08
BS9	140.92 ^cd^ ± 0.93	13.38 ^b^ ± 0.08
BS12	145.08 ^e^ ± 0.31	13.53 ^bc^ ± 0.04
BR3	137.04 ^bc^ ± 0.42	13.62 ^c^ ± 0.03
BR6	140.43 ^cd^ ± 1.84	13.68 ^cd^ ± 0.14
BR9	144.86 ^e^ ± 0.52	13.71 ^cd^ ± 0.04
BR12	150.33 ^f^ ± 0.49	13.89 ^d^ ± 0.03
BC3	135.04 ^ab^ ± 0.85	14.17 ^e^ ± 0.09
BC6	137.57 ^bc^ ± 0.41	14.34 ^ef^ ± 0.09
BC9	140.58 ^cd^ ± 0.94	14.98 ^f^ ± 0.04
BC12	144.62 ^e^ ± 1.04	15.61 ^g^ ± 0.08

Values are expressed as means ± standard deviation (*n* = 3). Different capital letters in a column indicate statistically significant differences among raw materials (*p* ≤ 0.05); different lowercase letters indicate significant differences among bread samples, according to Tukey’s test. CON—control bread; BS—breads with Lion’s Mane; BR—breads with Reishi; BC—breads with *Cordyceps*.

**Table 2:** Digestible starch, resistant starch and starch digestibility of raw materials and wheat bread enriched with medicinal mushroom powders.

Sample	Digestible Starch (%)	Resistant Starch (%)	Starch Digestibility (%)
		Raw materials	
Lion’s Mane	9.36 ^B^ ± 0.12	3.26 ^C^ ± 0.08	74.17 ^B^ ± 0.42
Reishi	15.84 ^C^ ± 0.19	6.25 ^D^ ± 0.09	71.71 ^A^ ± 0.37
Cordyceps	2.26 ^A^ ± 0.06	0.91 ^A^ ± 0.03	71.29 ^A^ ± 0.45
Wheat flour	59.55 ^D^ ± 0.31	1.10 ^B^ ± 0.04	98.19 ^C^ ± 0.12
		Bread samples	
CON	72.71 ^f^ ± 0.71	0.80 ^a^ ± 0.06	98.91 ^g^ ± 0.07
BS3	68.64 ^ef^ ± 0.76	1.12 ^ab^ ± 0.04	98.39 ^fg^ ± 0.04
BS6	67.24 ^def^ ± 0.65	1.35 ^ab^ ± 0.05	98.03 ^ef^ ± 0.06
BS9	66.11 ^cde^ ± 0.67	1.36 ^b^ ± 0.03	97.98 ^ef^ ± 0.02
BS12	64.13 ^bcde^ ± 0.58	1.38 ^bc^ ± 0.03	97.89 ^ef^ ± 0.04
BR3	65.43 ^bcde^ ± 0.73	1.93 ^cd^ ± 0.18	97.13 ^d^ ± 0.24
BR6	62.08 ^bcd^ ± 0.43	2.46 ^d^ ± 0.11	96.19 ^c^ ± 0.14
BR9	60.40 ^ab^ ± 0.73	3.66 ^e^ ± 0.24	94.29 ^b^ ± 0.29
BR12	55.98 ^a^ ± 0.58	4.22 ^f^ ± 0.21	92.99 ^a^ ± 0.31
BC3	71.83 ^f^ ± 0.69	1.21 ^ab^ ± 0.03	98.34 ^fg^ ± 0.02
BC6	68.21 ^ef^ ± 0.47	1.23 ^ab^ ± 0.11	98.23 ^f^ ± 0.15
BC9	65.83 ^bcde^ ± 0.58	1.37 ^b^ ± 0.10	97.96 ^ef^ ± 0.13
BC12	61.42 ^abc^ ± 0.83	1.54 ^bc^ ± 0.13	97.55 ^de^ ± 0.16

Values are expressed as mean ± standard deviation (*n* = 2). Uppercase superscript letters indicate significant differences (*p* ≤ 0.05) among raw materials, whereas lowercase letters refer to significant differences among bread samples (Tukey’s HSD test). CON—control bread; BS, BR and BC—wheat breads enriched with Lion’s Mane; Reishi and Cordyceps, respectively.

## Data Availability

The original contributions presented in this study are included in the article. Further inquiries can be directed to the corresponding author.
